# Bridging Knowledge Systems: A Community-Participatory Approach to EcoHealth

**DOI:** 10.3390/ijerph182312437

**Published:** 2021-11-26

**Authors:** Ann Marie Crosse, Margaret M. Barry, Mary Jo Lavelle, Jane Sixsmith

**Affiliations:** Health Promotion Research Centre, Discipline of Health Promotion, National University of Ireland Galway, H91 TK33 Galway, Ireland; a.crosse1@nuigalway.ie (A.M.C.); margaret.barry@nuigalway.ie (M.M.B.); jane.sixsmith@nuigalway.ie (J.S.)

**Keywords:** ecohealth, community, participation, ecological determinants, mapping, Ireland

## Abstract

Earth’s life-supporting ecosystems are integral to human and planetary health. Ecosystem services connect ecosystem functions to human wellbeing. The complex, multifaceted socio-ecological challenges of ecosystem decline necessitate a transdisciplinary approach, including the active and meaningful engagement and participation of local communities. Communities uniquely possess expert local knowledge, which, when integrated into policy development and community planning, has the potential to enhance and sustain ecosystem benefits for health and wellbeing. Community-informed mapping tools provide an opportunity for integrating science, policy, and public participation in data collection. However, there is a dearth of community-informed mapping tools demonstrating the interconnection of the ecological and social determinants of health at a place-based level. This paper presents a study that employs a community-based participatory research approach to mapping local knowledge systems on EcoHealth. The study seeks to develop a community mapping tool for shared dialogue and decision-making on EcoHealth between local communities and policymakers. The participatory research methods used to explore community awareness and knowledge regarding ecosystem services, health, and sustainability in the local area are described. The process of co-producing a Community EcoHealth Toolkit, based on the integration of different knowledge systems into local policy and planning, is discussed.

## 1. Introduction

The Earth’s life-supporting ecosystems are crucial for human and planetary health [1,2]. Health impacts from global environmental change (including climate change, ocean acidification, land degradation, water scarcity, overexploitation of fisheries, and biodiversity loss) pose serious adverse challenges to human health [1,3,4]. With increasing ecosystem degradation and ecological decline, the distribution of the health impacts is inequitable [5] and is widening inequalities in power, wealth, and access to resources [6]. This inequity presents a further threat to population health, and it is influenced by social as well as ecological determinants. These complex, multifaceted challenges to population health necessitate a comprehensive approach across sectors with multiple stakeholders, including the active, meaningful engagement with, and participation of, communities. Communities are in a unique position possessing expert local knowledge, which, when integrated into policy development and community planning, has the potential to enhance and sustain ecosystem benefits for health and wellbeing. This paper contextualises working with communities using an EcoHealth approach. A case study illustrates the use of community-based participatory research in the co-production of a Community EcoHealth Toolkit. The toolkit will establish key pathways to integrate ecological determinants of health and EcoHealth into policy and planning at a local community level.

### 1.1. Ecosystem Services and Health

Promoting health and wellbeing is improbable without an understanding of the need to conserve planetary biodiversity and ecosystems services. Ecosystems services are the tangible and intangible benefits that ecosystems provide to human beings, including regulating services (such as clean water, regulation of floods, soil erosion, pollination services, and disease outbreaks), as well as cultural services (including recreational and spiritual benefits, reflection, and creative and aesthetic experiences) [7,8]. Ecological determinants of health need to be regarded with similar importance as the social determinants of health in research, practice, and policy, for example, policy within natural resource management and public health [1,2,9,10]. To date, research relating to health and wellbeing has focused primarily on cultural and regulating services, predominantly in the areas of tourism, green and blue exercise, mental health, and climate change [11].

Numerous frameworks and regional guidance documents acknowledge the interconnection between planetary and population health; for example, The UN Agenda for Change 2030 and the Sustainable Development Goals [12]; Convention on Biological Diversity and Aichi Targets [13], Shanghai Declaration for Health Promotion [14]; UN Global Ecosystem Assessment [15]. Despite this, significant gaps remain in terms of implementation guidance and practice concerning the ecological determinants of health. Greater integration of the ecological determinants of health into public planning and policies is required [2,16], necessitating the development (from multiple perspectives) of a systematic shared understanding of ecosystems for population health. Systems change at all levels of governance is needed to promote and realise the potential contribution of healthy ecosystems for health. These two factors, shared understanding and multi-level systems changes, highlight the integral role of communities in supporting ecosystems for promoting population health [17,18]. The important role of public participation cannot be underestimated [19,20]. Public participation and community engagement are vital as communities possess the unique knowledge needed to inform effective, sustainable EcoHealth promotion and for that promotion to be scalable from a local to a national context. At the national level in Ireland, for example, public participation is recognised as fundamental in relation to sustainability indicators addressing local and national strategic sustainability priorities [19].

### 1.2. Ecosystem Services and Health Mapping

International and national policy in the areas of ecosystems and biodiversity, health promotion, climate change and sustainable development highlight the importance and potential for synergistic working and monitoring by demonstrating the environmental and societal conditions in each area [19]. Hence, there is a recognised need to identify mapping and measurement of interlinked socio-ecological data with identified criteria necessary to ensure credibility and spatial application across ecosystems and communities. Despite the call for community understanding of the valuation of ecosystem services, there is a dearth of community-informed mapping tools to demonstrate the interconnection of the ecological and social determinants at the place-based level. Community-informed mapping tools provide an opportunity for integrating science, policy, and public participation in data collection [21,22].

A series of ecosystems service frameworks and conceptual models for implementation captures the synergistic concepts and outline ecological, health, social and economic interconnections. Although not designed specifically to represent environment and health, these frameworks and conceptual models provide a potential resource for linking environment and health and positioning themselves across policy sectors [23]. For example, the UNEP Millennium Ecosystem Assessment [7] outlined four key services for health, whilst the Cascade Model [24] and Beyond the Cascade Model [25] describe the flow and stages between ecosystem services and societal benefits. These latter models were used in conjunction with the Common International Classification of Ecosystem Services System (CICES) [26] and environmental and economic valuation [27,28,29]. The IPBES Nature’s Contribution to People Framework builds on ecosystem service concepts and highlights the need to include both wellbeing valuations as well as indigenous and community knowledge systems and worldviews [30].

Building on the principles of health promotion, ecosystem management and sustainable development, EcoHealth is a promising transdisciplinary approach to understanding and managing the complex interconnectedness of ecosystem health, population health, and sustainability. The transdisciplinary nature of EcoHealth enables meaningful integration of multiple knowledge systems, with communities being explicitly identified as equal partners. Active community engagement in EcoHealth has the potential, through the development of a shared language, to bridge knowledge systems between communities and policymakers.

### 1.3. EcoHealth

EcoHealth is a conceptual movement, scientific area, and political endeavour. It is based on a broad, holistic positive understanding of health, with human health dependant on the environment and synergistic interactions between the two influenced by socio-ecological factors [31,32]. This important conceptual paradigm is used interchangeably with One Health and Planetary Health [33,34]. Nevertheless, from a socio-political perspective, health professionals and researchers perceive the paradigms differently [33]. In a review by Harrison et al., [32], drawing on earlier work by Charron [31] and Lebel [35], the principles of EcoHealth are identified as mirroring those of systems theory, sustainability, and knowledge-to-action, building on the three pillars of gender and social equity, transdisciplinary and participation [36,37]. The system-based approach of EcoHealth occupies the interface between social and ecological determinants of population and planetary health and positions itself to address determinants that influence the health and wellbeing of humans, natural systems, and sustainability [31,38]. This complexity requires a transdisciplinary approach to research, drawing on a wide range of disciplines [39], with participation from multiple stakeholders, such as policymakers, environmental management, and public health practitioners, and with communities identified as equal partners in the process [40]. The EcoHealth paradigm links public health and health promotion to natural resource management and sustainable development within an ecosystem approach to human health and biodiversity [40]. The inclusion of community knowledge systems is fully recognised within EcoHealth, and therefore, community engagement and participation are integral to EcoHealth practice.

### 1.4. Community Participation

Community participation is recognised as a critical strategy in enabling people to gain control over their health and address the determinants of community health and wellbeing. Despite the number of conceptual models that were developed to support research and practice in this area, there is a lack of conceptual clarity in the literature concerning the meaning of participation [41]. The seminal work is that of Arnstein [42], who developed a ladder of participation, which clearly delineates tokenistic approaches to participation from those of citizen control. More recently, a spectrum of public participation was developed [43], which defines the public’s role in any public participation process. It moves from “inform”, which is described as having a “promise to the public to keep them informed”, through processes such as consult, involve, collaborate, and empower, where empowerment “promises to implement the public’s decision”. Participation in EcoHealth actions is located at the empowerment end of the spectrum. The language of participation and community engagement is often used interchangeably [43]. Nevertheless, Ross, Baldwin, and Carter [44], acknowledging the inter-relatedness of the concepts, make a case for differentiation in the use of these terms in environmental decision-making. They contend that community engagement is a broader concept than that of participation and one that emphasises the participation of communities in decision-making planning, implementation, evaluation, and governance activities through consultation, collaboration, and community control. Community engagement is a longer-term activity and one that focuses on the relationship between engaged communities and other stakeholders [44]. A short scale of community engagement was developed for health research that begins at the level of consultation, with collaboration and community control [45].

Reviews and meta-analysis of community engagement research for health provide evidence that community engagement interventions across diverse areas, although limited in relation to environmental health, have a positive impact on a range of health outcomes [46,47,48]. Despite a lack of consensus on community engagement definitions [49], the core components are relevant to EcoHealth research and practice. These components are orientated to a broader conceptualisation of health and anchored at the collaborate and empowerment end of the participation spectrum. The lack of conceptual clarity in the terminology demonstrates the importance of having a shared understanding for community engagement and participation in activities that promote EcoHealth in both practice and research, which is especially relevant in transdisciplinary research with communities.

From the literature, Lerner and Berg [40] identify that participation for EcoHealth is by consensus and is based on cooperation and strives for action. Reference to an action orientation demonstrates the inter-connectedness of community engagement with the other characteristics and principles of EcoHealth, specifically “knowledge to action”, which in turn contributes to sustainability. Harrison et al. [32], drawing on Lincoln, Lynham, and Guba [50], identified the “constructivist leanings” of EcoHealth, which recognises that there are multiple realities that are socially constructed through engaging with the research process [50]. This supports the need for a participatory approach to research with value placed on the contribution of community knowledge. The constructivist leaning towards the EcoHealth worldview was supported in practice by a small study with Canadians from academic settings [51]. In a multi-disciplinary environment, this ontological perspective provides a common foundation to support coherence in research and practice.

Based on EcoHealth’s “constructivist leaning” foundation and aligning with identified EcoHealth principles [31] and Lebel’s [52] methodological pillars, the research presented in the illustrative case study uses a community-based participatory research methodology. With reference to the literature, Jagosh and colleagues [53] described community-based participatory research as an approach where “researchers and community stakeholders form equitable partnerships and co-construct research for the mutual and complementary goals of community health improvement and knowledge production” (p2). In this research study, the community is perceived as a critical stakeholder to work with, who holds community knowledge, as well as having a sense of place. In other words, the community is perceived as being the population that lives, works, and plays within the research landscape.

## 2. Methods and Materials

### 2.1. Community-Based Participatory Research

Many conceptual models of community-based participatory research (CBPR) have been developed. In particular, one that has been comprehensively and rigorously designed, implemented, and evaluated is that by Wallerstein and colleagues [54]. Envisioned as a dynamic tool for partnership working that is adaptable to implementation contexts [55], it comprises four interrelated domains with feedback loops between them. The domains are context, partnership and processes, intervention and research, and outcomes. A scoping meta-review of community-engaged research, using as an analytic structure the four domains of this conceptual model, reported on 100 reviews [55]. The context was identified as a dynamic concept with a focus in the literature on social structural and health issue importance. A thorough evaluation of the context was identified as a facilitator to collaborative success. A common conceptualisation of partnership was identified across the literature with the inclusion of power sharing. The research and intervention domain identified community engagement throughout the research process as contributing to outcomes. Fifty-five studies included in this review reported evidence of positive outcomes. This literature was found to focus mainly on health as the primary outcome, which is limiting as it does not capture all potential positive outcomes, although a broader notion of outcomes is conceptualised in the model with differentiation of those that are intermediate and long-term. Considering the findings across the domains, it is evident that the role of a truly engaged and empowered community in the research process (i.e., as equal partners in the process of knowledge production, problem definition, decision-making, implementation, evaluation, and dissemination) is integral to success and is directly relevant to research on EcoHealth. 

The EcoHealth and health promotion literature, particularly in relation to community engagement and the use of models of community-based participatory research in practice, provides the framework for the current research. The following case study illustrates this approach, drawing on the principles of community participation for empowerment [56], fostering socio-ecological knowledge in practice through co-learning [57], and capacity development and community ownership [58] for sustainability.

### 2.2. Case Study: A Community Participatory Research Approach to Mapping EcoHealth Knowledge in Local Communities 

This section presents the details of a study being conducted in two communities in the Republic of Ireland. This study employed a community-based participatory research approach to mapping local knowledge systems on EcoHealth and creating a community tool for shared dialogue and decision-making between local communities and policymakers. A participatory research approach is applied to explore community awareness and knowledge regarding ecosystem services, health, and sustainability in the local area. This research process was designed to inform the development of a novel Community EcoHealth Toolkit that can be used to map local knowledge so that it can be integrated into policy development and community planning to enhance ecosystem benefits for health and wellbeing.

This study aligns with current national health and environmental policy priorities concerned with advancing health-promoting environments and sustainable development in Ireland [59,60,61]. The study seeks to engage local community stakeholders in co-producing a Community EcoHealth Toolkit for mapping ecosystem services, health, and sustainability in the local area. The study applies a community participatory research approach to investigate the integration of local knowledge concerning the ecological determinants of health into policy and planning at a local community level. This will be accomplished through the co-production of a Community EcoHealth Toolkit, based on the use of scenario-visioning workshops and community participatory processes. The objectives of this study are as follows:

(1) To examine community awareness, knowledge, values, and understanding of the interconnection between population health, ecosystem services, and sustainability;
(2) To investigate community challenges and opportunities in relation to integrating the ecological determinants of health and the development of community health planning;
(3) To identify the main themes and methods required to support the development of a community-based EcoHealth Toolkit;
(4) To establish key pathways to integrate EcoHealth into policy and planning at a local community level.

#### 2.2.1. Study Design

The method employed in this study will be developed using, as a foundation, the ethos, and principles of EcoHealth that informed the choice of a community-based participatory research design with the community as involved partners in the process. This will inform the development of the process of stakeholder engagement and methods of data collection. The Community-Based Participatory Research (CBPR) conceptual model [54,58] provides the overarching framework for the design of this study. While this approach has increasingly been used for engaging community members in policy development for health, equity, and social justice, its use in relation to EcoHealth is less well documented. Community members are engaged as collaborative research partners in this study, and participatory methods are used to explore the awareness and knowledge of community members and their understanding of the interconnection between ecological determinants of health, ecosystem services, and sustainability on a local scale. Alongside community participation, key stakeholders and decision-makers in the areas of health, environment, education, and sustainable development are also engaged to inform the development of a Community EcoHealth Toolkit that can be employed for local community planning and sustainable development.

**Figure 1:**
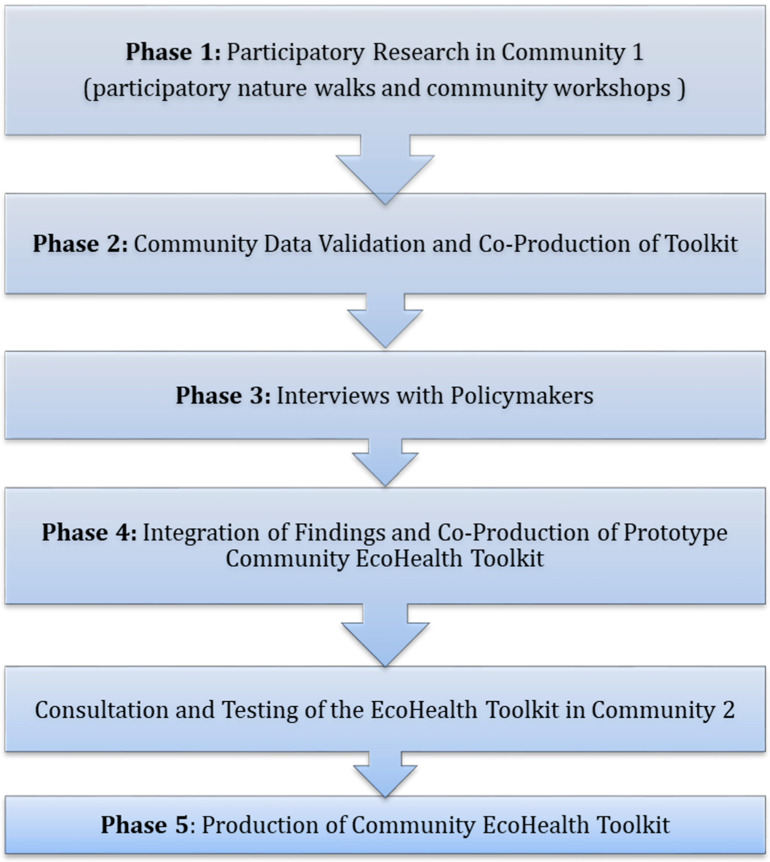
Phases of the Participatory Research Process.

In order to facilitate collaborative working with the local community, a local Community Forum was established comprising community groups, representatives, and key stakeholders from the local area. A smaller Steering Group, established to guide the research process in each community, comprised of key community stakeholders. Community researchers will be engaged as co-facilitators to help access and validate local knowledge and the history, nuances, and relationships surrounding local social, cultural, and ecological issues. The community co-facilitators will help to localise the research context, formulate culturally appropriate research questions and enable community ownership of the process [62]. Training workshops will be delivered in order to provide the co-facilitators with the grounding in the research process and the participatory methods of data collection (described in Section 2.4 and Section 2.5). Following a process of community engagement, the research will involve a number of key phases, as shown in Figure 1.

#### 2.2.2. Phase 1: Participatory Research-Nature Walks and Community Workshops

The first phase involved the use of participative methods of data collection with local community members. This stage of the fieldwork took place in Community 1 and consisted of guided nature walks with different groups of community members, followed by a series of interactive community workshops designed to explore levels of awareness, knowledge, and understanding of the ecosystem services, health, and sustainability within the local community.

#### 2.2.3. Phase 2: Community Data Validation and Co-Production of Toolkit

Phase 2 involves a series of community-wide workshops where the findings from Phase One are presented for validation and feedback by the community groups. The workshops will include individuals who participated in Phase 1 data collection, as well as being open to all other community members. Preliminary data analyses will be presented. Community groups will be engaged in reviewing the initial findings in order to consider and reflect on the emerging themes. Scenario forecasting and visioning methods will be employed in community workshops to provide valuable insight for further scenario iterations. This participatory co-production process, together with input from the local Steering Group, will promote and facilitate agreement and decision-making on the key themes, methods, and materials that need to be incorporated into the Community EcoHealth Toolkit. A fundamental element of a participatory co-production process is that it promotes reflection and learning about EcoHealth problems amongst its community participants, which may then be translated into action beyond the remit of the research study.

#### 2.2.4. Phase 3: Interviews with Policymakers

A series of qualitative interviews will be conducted with policymakers working in the areas of health and wellbeing, environment, sustainability, climate change, and planning. These interviews are designed to explore the interconnections between ecosystem services and community health and are informed by the emerging themes from the community workshops.

#### 2.2.5. Phase 4: Integration of Findings and Production of Community EcoHealth Toolkit-Consultation and Testing of the EcoHealth Toolkit in Community 2

Following the integration of research findings from Phases 1 to 3, a prototype Community EcoHealth Toolkit will be developed, reviewed by the Steering Group, and tested with community members and decision-makers in Community 2. The findings from Phases 1–3 will inform the selection of the toolkit mapping methods and processes for implementation, assessing community-based knowledge and awareness and understanding of the ecological determinants of health at a local place-based level. The prototype EcoHealth Toolkit will be tested in Community 2, where a second community Steering Group will be established to oversee and verify the findings.

#### 2.2.6. Phase 5: Production of a Community EcoHealth Toolkit

Community 1 and Community 2 Steering Groups will be actively involved in developing the final draft of the EcoHealth Toolkit. The Community EcoHealth Toolkit will include a guidance document to facilitate the effective communication of local knowledge and its integration into local and regional decision-making processes on community health and local ecosystem management with governmental and non-governmental agencies.

### 2.3. Settings and Participants

Two rural communities were selected for participation in this research study. Rural communities and economies are integral to environmental wellbeing and development and are pivotal in shaping a healthy and sustainable future. They play a central role in the current and future provision, maintenance, and restoration of Ecosystem Services. As gatekeepers of the landscape, they are well placed to explore this interdependence, contribute to shared learning, and reframe the narrative towards planetary and population health [60]. The selection of the two case study areas was informed by a research gap identified in the literature concerning the need to address understudied cohorts, such as older people and youth, and habitats associated with inland waterways and wetlands [63].

The two areas were selected based on the lead researcher’s working knowledge of the communities, both of which have strong community-based platforms with access to key community stakeholders and an interest in the research topic. From an environmental perspective, the geographical areas selected comprise a wide range of natural habitats (including coastline, fields, mountains, bog, meadow, river and streams, woodland, farmed and fished areas). This enables a range of ecosystem services to be integrated into the research. Community 1, a coastal area with a population of 2120 people, was the location for Phase 1 of the initial fieldwork. Community 2 is an inland community with a population of 3000 persons. This is where, in Phase 4, the Community EcoHealth Toolkit prototype will be tested in consultation with local community stakeholders. The production of the final toolkit takes place in Phase 5 of the research fieldwork.

A population-based approach with a focus on community inclusion enables an exploration of diverse and varying levels of awareness, knowledge, and understanding of the interconnection between place and people. Purposive samples of community members will beinvited to participate in the research process in both communities. The participants will be drawn from across the life-course, from school-going children and local youth groups to adult groups and older people and members of the wider Community Forum. The research identified certain life course cohorts as being understudied, such as older people and youth [17]. In addition, purposeful samples will be drawn from those working directly on land and sea, for example, farmers and fishermen whose livelihood and wellbeing depend on healthy functioning ecosystems [64], and those engaged in nature-based activities such as walking groups and environmental groups who may have a vested interest in green exercise, environmentalism, and ecotourism. Policymakers and state sectors will be purposively selected to participate in the research based on their active involvement at a policy level in health promotion, environment, sustainable development, and county planning. These sectors are key influencers in any action that is based on the interconnection between the ecological and social determinants of health.

### 2.4. Participatory Methods of Data Collection

Development of the research process entails the use of a range of qualitative participatory methods of data collection, selected to explore the multidimensional and complex issue of EcoHealth and to balance an exploratory, engaging, and empowering process with consciousness-raising, critical reflection, and priority setting. The selection and development of research methods were informed by the theoretical underpinnings of community participatory research, drawing on the Community-Based Participatory Research (CBPR) conceptual model [65], as well as the principles of EcoHealth.

**Figure 2:**
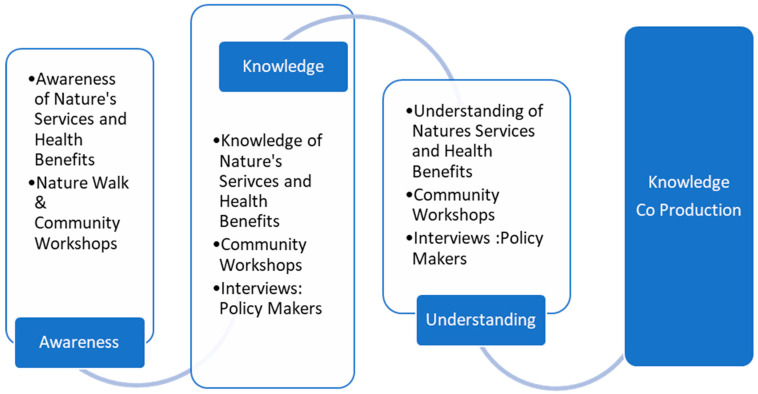
Research Process of Knowledge Co-Production.

The research methods of participatory data collection identified from the research literature were chosen to capture the emergent process of knowledge co-production gained from multi-phased group discussions and dialogue. Open questions exploring levels of awareness and knowledge are designed to support a re-connection with a local place, as well as the generation of new ideas, leading to knowledge production [62,66]. Emerging concepts and issues were examined and analysed. Scenario-type questions were employed to enable a deeper understanding of the opportunities and challenges of integrating the ecological determinants of health into community planning and local policy development. The multi-phased iterative process of knowledge co-production, which is outlined in Figure 2, informs the development of the Community EcoHealth Toolkit.

### 2.5. Participatory Methods

Among the research tools employed to support this iterative participatory process are outdoor nature walks and indoor “nature in place” stations to explore the connection to place, community mapping, and place valuation from the perspective of the community and ranking and scoring methods to compare preferences, priorities, and opinions on various EcoHealth issues [67]. Timelines will be employed to explore trends in the ecological and social changes in the local area and their impact on ecosystem alteration. The use of these participatory methods, reflective discussion, and critical dialogue was designed to support a deeper reflection and analysis of the community understanding of the interdependence between community health, ecosystem services, and the natural environment. The participatory methods enable critical reflection on the synergies, co-benefits, and trade-offs that need to be considered within local community planning and the potential links between community health and sustainable development. The critical dialogue will connect knowledge systems and generate new insights into the links between local places, ecosystem services, and health. This knowledge enriches the next phase of the research process and informs the main themes to be included in the Community EcoHealth Toolkit. The methods to be employed, as shown in Table 1, are briefly described.

Outdoor nature walks were employed to explore the level of awareness, knowledge, and understanding of the health benefits derived from the local ecosystem. A facilitator guided a local group on a half-hour walk within 5 km of their home, covering a variety of habitats. The nature walk enabled participants to base themselves in nature and to explore the connections between self and place [68]. Walking interviews were designed to produce a spatial and locational discourse of place (which is structured geographically) that enables detailed insights into the meanings and practices people associate with their environment [69]. Within this format, the participants were regarded as experts in their geographical area [70]. While on the walk, participants were encouraged to actively engage with nature through the senses of sight, smell, touch, and sounds, provoking memories and experiences linked to a local place. Lauwers and colleagues [69] posit that this method challenges the interviewer and the interviewee to delve deeper into topics raised along the walk as both parties encounter new features to discuss. They were also encouraged to use audio and photography to record their thoughts, collect nature features, and record their reactions and feelings. Mapping the data from these interviews produces a narrative that unfolds through a place, organising experiences spatially rather than temporally [69]. A semi-structured interview was also completed by the participants that explored place awareness, sense of place, knowledge of local habitats, and understanding of place-based health and wellbeing benefits. A participative group reflection was facilitated on completion of the nature walk to explore community perceptions of the benefits of ecosystem services, as well as how shared social and cultural values shape the interaction with and experience of the natural environment. The half-hour nature walk and short reflection were designed to increase participants’ insights and awareness of their own knowledge and to encourage greater sharing within the follow-on community workshop. The different manifestations of knowledge include information, knowledge of local places, as well as personal experiences, events, memories, and connections.

Community workshops are designed to facilitate participants in exploring further the links between local places, earth systems, ecosystem services and community health, deepening discussions through consciousness-raising, reflection and analysis, and collating and theming data. The workshops will comprise of discussion-generating questions supported by a range of participatory tools to support sequential learning and reflection. The qualitative themes will be presented in subgroups and coupled to a member check validation by asking participants to provide input on whether the themes accurately reflected their experiences. The workshops will be co-facilitated by the researcher and the community facilitators. Emphasis will be placed on creating a shared and inclusive space where different points of view can be encouraged, articulated, listened to, and discussed. The collective knowledge on EcoHealth elicited through this process will then be used to inform the constituents of the community-mapping EcoHealth Toolkit in Phase 4.

Research on Phase One is currently being completed and analysed, following which the validation process at the community level will be undertaken. Levels of understanding are documented throughout the process to ensure that new knowledge systems can contribute and inform policy and decision making and enable integration of the community and policy knowledge systems into the development of the Community EcoHealth Toolkit. The results of the community co-production research process facilitate an understanding of the benefits, impacts, and synergies of integrating community understandings of the ecological determinants of health into local community planning and sustainable development.

### 2.6. Phase 1 Preliminary Findings

This section outlines some preliminary findings from a thematic analysis of data collected in Phase 1 of the research, which involved outdoor nature walks with community participants to explore the level of awareness, knowledge, and understanding of the health benefits resulting from the local ecosystem.

Six nature walks were conducted with 73 participants, who were drawn from five cohort groups: primary school children (*n* = 25); youth groups (*n* = 17); walking and environmental groups (*n* = 9); farmers (*n* = 12); and older people (*n* = 10), all resident in the local community. Of the 73 individuals who participated, 56% were female (*n* = 41), and participants ranged in age from 10 to 89 years. The nature walks took place in a variety of habitats: woodland, hedgerow, wetland, and beach. Following the interviews and short reflections, a cyclical process of thematic data analysis was undertaken, which involved transcribing, coding, and categorising the data, identifying and linking emerging themes. This process was completed with the research co-facilitator. Preliminary results are presented, which provide an overview of the key themes emerging from this stage of the research study.

The main themes emerging on the interaction with nature during the walks included the enjoyment of exploration, sense of place and belonging, feelings of calm, happiness and invigoration, memories of childhood, and knowledge of nature. As the interview questions explored perceptions of place-based health and wellbeing benefits, the emerging themes were found to reflect all four of the ecosystem services. Cultural, regulating, provisioning, and supporting services were all recognised within the responses, with the most frequently cited being cultural and regulating services in the form of mental, emotional, social, and physical health benefits. Least recognised were provisioning and supporting services in the form of goods from the sea and healthy soil.

All participants (100%) across the age cohorts clearly identified key features of healthy places as being rich and diverse in nature and biodiversity, which underlie all ecosystem services; “*Totally healthy-an abundance of nature-so many different trees, flora, fauna*”. Older respondents demonstrated an understanding of the need to conserve and protect nature for human and ecosystem health and wellbeing; “*Reduce land clearance and tree felling, Designate protected areas–signage to educate people, develop woodland corridor for wildlife in {County Name}, create protected marine zone in Lough {Name}*” (Older Person). All cohorts (100%) recognised mental and emotional health benefits such as relaxation, happiness and reduced stress. Younger participants referred to nature being a place of escapism: “*improves your mental health as you can take a break from everything*”; “*Its calming and away from the real world*” (youth); while participants in the older age cohorts recognised broader health benefits citing “*Exercise, fresh air and scenery benefits your wellbeing, mentally, physically and psychologically*” (Older Person). All age groups (100%) referred to a feeling of emotional wellbeing when immersed in nature. Awareness of nature and nature’s health benefits appeared to be increased through sensory exploration: “*the sea, it has nice sound*”; “*I like the waves crashing*” (School Child); “*Lovely countryside with water running in streams, birds singing, smells of mossy earth and various natural flowers in our habitat*” (Older Person). All cohorts (100%) recognised physical health benefits including, exercising within natural spaces (green exercise) and an abundance of fresh unpolluted air: “*Exercise, fresh air and scenery benefits your wellbeing, mentally, physically and psychologically*”; “*Air ….healthy, clean, pure and fresh*”; “*wonderful fresh air*” “*with trees providing pure oxygen*”. Younger age cohorts recognised the health benefits of swimming as blue exercise. The potential of developing Nature Services for health was primarily identified by older age groups, mainly in relation to cultural and regulating services such as accessible walkways: “*walks created along the river*” and developing pathways through forests to promote the health benefits of trees in providing oxygen. Participants across all age cohorts (100%) identified the social benefits of being outdoors. Younger age groups referred to fun with friends and family, while older age groups referred to sharing memories linked to cultural heritage and sense of place.

These findings indicate that the nature walks facilitated exploration of community perceptions on the benefits of ecosystem services, as well as providing insights into how shared social and cultural values shape the interaction with and experience of the natural environment. The use of the walking interview and the participative group reflection worked well in stimulating participants’ insights and awareness of their own knowledge and encouraged sharing among the groups. The different manifestations of knowledge included information, knowledge of local places, as well as personal experiences, events, memories, and connections. The Phase 1 nature walk findings provide a platform from which to explore further the factors which influence different levels of community awareness, knowledge, and understanding of the social and ecological interconnections of health at the place-level.

Adaptions to the initial data collection procedures were made in order to adhere to COVID-19 pandemic restrictions. Phase 1 of the research process was, therefore, limited to outdoor nature walks to date. The follow-up community workshops in Phase 1 are scheduled to be completed in December 2021, as restrictions on in-door meetings have been reduced.

## 3. Conclusions

EcoHealth is an emerging area of research and practice, which provides a framework for cross-sectoral collaborative working between health, environment, sustainable development, and local communities. EcoHealth provides conceptual, principled, and procedural place-based approaches and guidance to catalyse a transformation toward public health equity and social justice for future populations. It repositions health and environment sectors towards integrated proactive and sustainable policies and creates a negotiated space for synergistic working between communities, practitioners, and decision-makers. This negotiated space needs to be embedded in a common language, based on the interconnections between ecosystem services, health, and sustainable development. The bridging of knowledge systems requires enabling processes for the exchange of multiple forms of knowledge. Active engagement with local communities is vital to this process, enabling knowledge-sharing processes that are equitable, diverse, and empowering, respecting the integrity of each knowledge system. This requires community-led mobilisation of knowledge, hearing different perspectives and understandings, and translating the knowledge systems into application and learning for transdisciplinary working [71]. This also requires the capacity to communicate effectively so that the true values of biodiversity and ecosystem functions, services, and benefits are understood, embraced, and translated into policy.

This paper outlines how an EcoHealth approach provides a conceptual framework to explore the association between ecosystems, health, and sustainability in the local place. Community-based participatory research, which aligns with EcoHealth principles, is employed to facilitate working with communities as equal partners in order that local knowledge can be integrated into the research, practice, and policy processes. Within this study, community participatory processes are structured to enable and empower community members to critically explore levels of awareness, knowledge, and understanding of nature services and health benefits. Through a process of group dialogue and reflection, communities have an opportunity to develop a shared perspective, claim community expert knowledge, and participate in shared decision making. The results of the community co-production research process in this study will facilitate an understanding of the benefits, impacts, and synergies of integrating community knowledge of the ecological determinants of health into local community planning and sustainable development.

The use of participative models of engagement is integral to any discussion or action related to the health of the planet and its population. Communities possess expert local knowledge that needs to be integrated into policy development and community planning to enhance and sustain ecosystem benefits for health and wellbeing. Hence, community-informed mapping tools provide an opportunity for integrating science, policy, and public participation in data collection. This paper outlined an ongoing research study that demonstrates the use of a participatory approach to the co-production of a Community EcoHealth Toolkit designed to map local knowledge for shared dialogue and decision-making on EcoHealth between communities and policymakers. The importance and timely nature of such community-based research cannot be underestimated if we are to achieve a deeper understanding of how to manage the complex interconnectedness of ecosystem health, population health, and sustainability.

## Figures and Tables

**Table 1:** Participatory Methods to be Employed Across the Research Process.

Research Phase	Participatory Methods	Community Cohort	Number of Participants Per Workshop
Phase 1	Outdoor Guided Nature Walk Questionnaire and Photo voice x 7	School Children Age 10–11 Teenagers, Older People Community Forum, Walking Group/Environmental Group, Farmers and Fishermen, Generic Community	100 Participants 7 Walks: 12–13 per walk
Phase 1	Community Group Workshop Place Stations Timeline Ranking and Scoring Thematic group discussions x 7 Groups	School Children Age10–11 Teenagers Older People Community Forum Walking/Environmental Group Farmers and Fishermen All other community members	100 Participants 7 Workshops: 12–13 per workshop
Phase 2	Community Group Workshop H Diagrams Timeline Ranking and Scoring Scenario Forecasting Thematic Group Discussions	Representatives from Phase 1 workshops/ Community Forum and All other community members	90 Participants 3–4 Workshops: 30 per workshop
Phase 3	Policy Makers Semi-structured Interviews	Health and Wellbeing, Environment Sustainable Development Climate Change Local Planning Department	5 Interviews: 5 people
Phase 4	Community Workshop	Co-facilitators and Representative from Community Forum	18 Participants 3 Workshops: 6 per workshop
Phase 5 Second Community in separate location.	Community Group Workshop Thematic Group Discussions using methods agreed in Toolkit	Community Forum and Generic Community	90 Participants 3–4 Workshops: 25–30 per workshop
Total			398 Participants 23 Community Workshops

## Data Availability

The data presented in this study comprises part of a PhD research project and hence, it is not publicly available at this time. Data access will be restricted until after the completion of the doctoral research study.

## References

[1] Intergovernmental Panel on Climate Change (IPCC) (2013). Climate Change 2013: The Physical Science Basis–Summary for Policy Makers. Contribution of Working Group I to the Fifth Assessment Report of the Intergovernmental Panel on Climate Change.

[2] Hancock T., Spady D.W., Soskoine C.L., Allison S., Chircop A., McKibbon G., Harper S., Parkes M., Poland B. (2015). Global Change and Public Health: Addressing the Ecological Determinants of Health—The Report in Brief.

[3] Whitmee S., Haines A., Beyrer C., Boltz F., Capon A., Ferreira de Souza Dias B., Ezeh A., Frumkin H., Gong P., Head P. (2016). Safeguarding human health in the Anthropocene epoch: Report of the Rockefeller Foundation-Lancet Commission on planetary health. Lancet.

[4] Diaz S., Settele J., Brondizio E.S., Ngo H.T., Guèze M., Agard J., Arneth A., Balvanera P., Brauman K.A., Butchart S.H.M., The Intergovernmental Science-Policy Platform on Biodiversity and Ecosystem Services (IPBES) (2019). Summary for Policymakers of the Global Assessment Report on Biodiversity and Ecosystem Services of the Intergovernmental Science-Policy Platform on Biodiversity and Ecosystem Services.

[5] Marmot M., Friel S., Bell R., Houweling T.A., Taylor S. (2008). Commission on Social Determinants of Health. Closing the gap in a generation: Health equity through action on the social determinants of health. Lancet.

[6] McMichael A.J., Nyong A., Corvalan C. (2008). Global Environmental Change and Health: Impacts, Inequalities, and the Health Sector. BMJ.

[7] Millennium Ecosystem Assessment (MEA) (2005). Ecosystems and Human Well-Being: Synthesis.

[8] (2011). UK National Ecosystem Assessment (UK NEA).

[9] Waltner-Toews D., Kay J. (2005). The Evolution of an Ecosystem Approach: The Diamond Schematic and an Adaptive Methodology for Ecosystem Sustainability and Health. Ecol. Soc..

[10] Parkes M., Panelli R., Weinstein P. (2003). Converging paradigms for environmental health theory and practice. Environ. Health Perspect..

[11] Pretty J., Peacock J., Sellens M., Griffin M. (2005). The mental and physical health outcomes of green exercise. Int. J. Environ. Health Res..

[12] United Nations (2015). Transforming Our World: The 2030 Agenda for Sustainable Development.

[13] United Nations (2010). Convention on Biodiversity 2011–2020 and Aichi Biodiversity Targets.

[14] World Health Organization (2016). Shanghai Consensus on Promoting Health in the 2030 Agenda for Sustainable Development.

[15] United Nations (2019). Global Assessment Report, Intergovernmental Science—Policy Platform on Biodiversity and Ecosystem Services (IPBES).

[16] Bunch M.J. (2016). Ecosystem Approaches to Health and Well-being: Navigating Complexity, Promoting Health in Social–Ecological Systems. Syst. Res. Behav. Sci..

[17] Carlin C., Kindermann G., Britton E., Cormican M., Domegan C., Gormally M., O’Donovan D. (2020). Nature and Environment to Attain and Restore Health (NEAR Health).

[18] Domegan C., Kindermann G., Brolcháin N.Ó., Britton E., Carlin C., Osagie E., O’Loughlin M., Cormican M., Donovan F., Mulcahy M. (2021). Our Environment, Our Health, Our Wellbeing: Access to Blue/Green Spaces in Ireland.

[19] Flood S., Dwyer N., Gault J. (2021). Report 379: Policy Coherence in Adaptation Studies: Selecting and Using Indicators of Climate Resilience.

[20] Fahy F., O Cinnéide M. (2009). Developing and testing an operational framework for assessing quality of life. Environ. Imp. Assess. Rev..

[21] Reis S., Morris G., Fleming L.E., Beck S., Taylor T., White M., Depledge M.H., Steinle S., Sabel C.E., Cowie H. (2015). Integrating health and environmental impact analysis. Public Health.

[22] Díaz S., Pascual U., Stenseke M., Martín-López B., Watson R.T., Molnár Z., Hill R., Chan K., Baste I.A., Brauman K.A. (2018). Assessing nature’s contributions to people. Science.

[23] Ford A.E., Graham H., White P.C. (2015). Integrating Human and Ecosystem Health through Ecosystem Services Frameworks. EcoHealth.

[24] Potschin M., Haines Young R. (2011). Ecosystem Services: Exploring a geographical perspective. Prog. Phys. Geogr..

[25] Potschin M., Haines Young R. (2016). Conceptual Frameworks and the Cascade Model. OpenNESS Ecosystem Services Reference Book.

[26] Haines-Young R., Potschin-Young M. (2018). Revision of the Common International Classification for Ecosystem Services (CICES V5.1): A Policy Brief. One Ecosyst..

[27] Crossman N.D., Burkhard B., Nedkov S., Willemen L., Petz K., Palomo I., Drakou E.G., Martin-Lopez B., McPhearson T., Boyanova K. (2013). Blueprint for mapping and modelling ecosystem services. Ecosyst. Serv..

[28] Gret-Regamey A., Alteegg J., Siren E.A., VanStrien M.J., Weibel B. (2015). Integrating ecosystem services into spatial planning. Landsc. Urban Plan..

[29] Constanza R., Kubiszewski I. (2012). The authorship structure of “ecosystem services” as a transdisiplinary field of scholarship. Ecosystem. Serv..

[30] Diaz S., Demissew S., Carabias J., Joly C., Lonsdale M., Larigauderie N., Adhikari A., Adhikari J.R., Arico S., Báldi A. (2015). The IPBES Conceptual Framework-connecting nature and people. Curr. Opin. Environ. Sustain..

[31] Charron D.F., Charron D.F. (2012). Ecohealth: Origins and approach. Ecohealth Research in Practice: Innovative Applications of an Ecosystem Approach to Health.

[32] Harrison S., Kivuti-Bitok L., Macmillan A., Priest P. (2019). EcoHealth and One Health: A theory-focused review in response to calls for convergence. Environ. Int..

[33] Roger F., Caron A., Morand S., Pedrono M., de Garine-Wichatitsky M., Chevalier V., Tran A., Gaidet N., Figuié M., de Visscher M. (2016). One Health and EcoHealth: The same wine in different bottles?. Infect. Ecol. Epidemiol..

[34] Zinsstag J., Jeggo M., Schelling E., Bonfoh B., Waltner-Toews D., Lelii S. (2012). Convergence of EcoHealth and One Health. Ecohealth.

[35] Lebel J. (2003). Health: An Ecosystem Approach: Focus.

[36] Checkland P. (1981). Systems Thinking, Systems Practice.

[37] Parkes M.W. (2011). Diversity, emergence, resilience: Guides for a new generation of Ecohealth research and practice. EcoHealth.

[38] Unahalekhaka A., Pichpol D., Meeyam T., Chotinun S., Robert G., Robert C., Ecohealth Manual (2018). EcoHealth–One Health Resource Centre, Chiang Mai University, Thailand. https://cgspace.cgiar.org/bitstream/handle/10568/33566/EcoHealthManual-ChiangMai.pdf.

[39] Zinsstag J., Waltner-Toews D., Tanner M., Zinsstag J., Waltner-Toews D., Tanner M. (2015). Theoretical Issues of One Health.

[40] Lerner H., Berg C.A. (2017). Comparison of Three Holistic Approaches to Health: One Health, EcoHealth, and Planetary Health. Front. Vet. Sci..

[41] South J., Bagnall A.M., Stansfield J.A., Southby K.J., Mehta P. (2019). An evidence-based framework on community-centred approaches for health: England, UK. Health Promot. Int..

[42] Arnstein S.A. (1969). Ladder of Citizen Participation. J. Am. Plan. Assoc..

[43] International Association for Public Participation (IAP2) (2018). IAP2 Spectrum of Public Participation, IAP2 International Federation. https://cdn.ymaws.com/www.iap2.org/resource/resmgr/pillars/Spectrum_8.5x11_Print.pdf.

[44] Ross H., Baldwin C., Carter R.W. (2016). Subtle implications: Public participation versus community engagement in environmental decision-making. Aust. J. Environ. Manag..

[45] Boote J., Telford R., Cooper C. (2002). Consumer Involvement in Health Research: A Review and Research Agenda. Health Policy.

[46] Anderson L.M., Adeney K.L., Shinn C., Safranek S., Buckner-Brown J., Krause L.K. (2015). Community Coalition-Driven Interventions to Reduce Health Disparities Among Racial and Ethnic Minority Populations. Cochrane Database Syst. Rev..

[47] O’Mara-Eves A., Brunton G., McDaid D., Kavanagh O.J., Jamal F., Matosevic T., Harden A., Thomas J. (2013). Community Engagement to Reduce Inequalities in Health: A Systematic Review, Meta-Analysis and Economic Analysis.

[48] Brunton G., Caird J., Stokes G., Stansfield C., Kneale D., Richardson M., Thomas J. (2015). Review 1: Community Engagement for Health via Coalitions, Collaborations and Partnerships—A Systematic Review.

[49] Sarrami-Foroushani P., Travaglia J., Debono D., Braithwaite J. (2014). Key concepts in consumer and community engagement: A scoping meta-review. BMC Health Serv. Res..

[50] Lincoln Y.S., Lynham S.A., Guba E.G., Denzin N.K., Lincoln Y.S. (2018). Paradigmatic controversies, contradictions, and emerging confluences revisited. The SAGE Handbook of Qualitative Research.

[51] Lisitza A., Wolbring G. (2018). EcoHealth and the determinants of health: Perspectives of a small subset of Canadian academics in the EcoHealth community. Int. J. Environ. Res. Public Health.

[52] Lebel J. (2004). Ecohealth and the developing world. J. EcoHealth.

[53] Jagosh J., Bush P.L., Salsberg J., Macaulay A.C., Greenhalgh T., Wong G., Cargo M., Green L.W., Herbert C.P., Pluye P. (2015). A realist evaluation of community-based participatory research: Partnership synergy, trust building and related ripple effects. BMC Public Health.

[54] Wallerstein N., Duran B., Oetzel J., Minkler M. (2018). Community-Based Participatory Research for Health: Advancing Social and Health Equity.

[55] Ortiz K., Nash J., Shea L., Oetzel J., Garoutte J., Sanchez-Youngman S., Wallerstein N. (2020). Partnerships, processes, and outcomes: A health equity-focused scoping meta-review of community-engaged scholarship. Annu. Rev. Public Health.

[56] Rifkin S.B., Pridmore P. (2001). Partners in Planning: Information, Participation, and Empowerment.

[57] Duboz R., Echaubard P., Promburom P., Kilvington M., Ross H., Allen W., Ward J., Deffuant G., de Garine-Wichatitsky M., Binot A. (2018). Systems thinking in practice: Participatory modeling as a foundation for integrated approaches to health. Front. Vet. Sci..

[58] Minkler M., Wallerstein N. (2008). Community Based Research for Health: Process to Outcomes.

[59] Government of Ireland, Healthy Ireland (2013). A Framework for Improved Health and Wellbeing 2019–2025.

[60] Government of Ireland (2021). Our Rural Future Rural Development Policy 2021–2025.

[61] (2017). Government of Ireland (2017) National Biodiversity Action Plan 2017–2021 Department of Culture. npws.ie.

[62] de Negri B., Thomas E., Illinigumugabo A., Muvandi I., Lewis G. (1998). Empowering communities: Participatory techniques for community-based programme development. Volume 1(2): Trainer’s Manual (Participant’s Handbook).

[63] Britton E., Kindermann G., Domegan C., Carlin C. (2020). Blue care: A systematic review of blue space interventions for health and wellbeing. Health Promot. Int..

[64] Macra (2020). Make the Moove Farmers Matter.

[65] Wallerstein N. (2020). Commentary on Community-Based Participatory Research and Community Engaged Research in Health for Journal of Participatory Research Methods. J. Part. Res. Methods.

[66] Chambers R. (1992). Rural Appraisal: Rapid, Relaxed & Participatory.

[67] Oxfam (2003). Making Waves in Walsall.

[68] Evan J., Jones P. (2011). The walking interview: Methodology, mobility and place. Appl. Geogr..

[69] Lauwers L., Bastiaens H., Remmen R., Keune H. (2020). Nature’s Contributions to Human Health: A Missing Link to Primary Health Care? A Scoping Review of International Overview Reports and Scientific Evidence. Public Health Front..

[70] Emmel N., Clark A. (2009). The Methods Used in Connected Lives: Investigating Networks, Neighbourhoods and Communities.

[71] Tengo M., Hill R., Malmer P., Raymins C.M., Spierenburg M., Danielsen F., Emlqvist T., Folke C. (2017). Weaving knowledge systems in IPBES, CBD and beyond-lessons learned for sustainability. Sci. Direct.

